# Wall Paper

**Published:** 1873-02

**Authors:** 


### WALL PAPER.

If a wall is repapered, the old paper ought to be first taken off, and the wall washed with strong lye water, and when the wall is cleaned and dried, lay on the new; for want of this precaution, members of various families have sickened and died.

Green wall paper, especially that showing a velvety green, has undoubtedly caused death in various places; if well varnished, every year or two, these injurious effects may possibly be prevented, because the varnish glues the poisonous particles to the wall, so as to prevent their being detached. At the same time, a plastered wall with a "hard finish," well washed and whitewashed every year, is the healthiest and the cleanest; no other kind should be slept in, if the sleeper has his choice; painted walls are next best, but papered walls, of whatever color, will hide dust and dirt, and absorb dampness, and, in proportion, will render the atmosphere of any room more or less impure.

